# Effects of pressure versus volume controlled ventilation on postoperative pulmonary complications during elderly trendelenburg laparoscopic surgery

**DOI:** 10.1038/s41598-026-55547-0

**Published:** 2026-06-22

**Authors:** Xiaoxi Liu, Zizuo Zhao, Qiurong Wu, Chaohang Luo, Chunya Xiao, Tao Luo, Bin Wang

**Affiliations:** Department of Anesthesiology, The First Affiliated Hospital of Chongqing Medical University, No.1 Youyi Road, Yuzhong District, Chongqing, 400016 China

**Keywords:** Postoperative pulmonary complications, Pressure-controlled ventilation, Volume-controlled ventilation, Laparoscopic surgery, Trendelenburg position, Elderly, Diseases, Health care, Medical research

## Abstract

The impact of different mechanical ventilation modes on pulmonary outcomes following laparoscopic surgery in the Trendelenburg position remains unclear. This study aimed to compare the effects of two common ventilation modes on postoperative pulmonary complications (PPCs) in elderly patients undergoing such procedures. Elderly patients scheduled for laparoscopic surgery in the Trendelenburg position were randomly allocated to receive either pressure-controlled ventilation (PCV) or volume-controlled ventilation (VCV). Both groups were managed with a lung-protective ventilation strategy. The primary outcome was the incidence of PPCs within the first three postoperative days. Airway pressures, details enabling the calculation of respiratory system dynamic compliance (Cdyn) and arterial blood gas levels were also recorded at predetermined intraoperative time points: before anesthesia induction (T0); 10 min after tracheal intubation in the supine position without pneumoperitoneum (T1); 30 min (T2) and 60 min (T3) after establishing pneumoperitoneum and the Trendelenburg position; and at the end of surgery after returning to the supine position (T4). Compared with the VCV group (32.1%), the PCV group exhibited a significantly lower incidence of PPCs (13.0%; χ^2^ = 5.758, *P* = 0.016) (RR = 0.403, 95% CI: 0.183–0.888). Furthermore, patients managed with PCV exhibited significantly lower intraoperative airway pressures—including peak airway pressure (Ppeak), plateau pressure (Pplat), and driving pressure (ΔP)—as well as reduced dead space fraction (VD/VT) and arterial partial pressure of carbon dioxide (PaCO₂). Cdyn was higher in the PCV group. In elderly patients undergoing laparoscopic surgery in the Trendelenburg position, pressure-controlled ventilation was shown to improve Cdyn and was associated with a lower composite rate of postoperative pulmonary complications than volume-controlled ventilation. Whether these physiological advantages translate into clinically meaningful benefit requires confirmation in larger studies.

## Introduction

Postoperative pulmonary complications (PPCs) are common after abdominal surgery and are associated with prolonged hospital stay and increased mortality^1^. Laparoscopic surgery in the Trendelenburg position is widely used for lower abdominal and pelvic procedures. However, carbon dioxide (CO₂) pneumoperitoneum adversely affects intraoperative respiratory mechanics^2^. The increase in intra-abdominal pressure displaces the diaphragm cephalad, resulting in reduced lung volumes, decreased functional residual capacity, increased airway resistance, impaired pulmonary compliance, and a reduced oxygen reserve^3^. These changes are further aggravated by the Trendelenburg position, which additionally compromises lung volumes and increases the risk of intraoperative hypoxaemia^4^. Moreover, the establishment and release of pneumoperitoneum might contribute to ischaemia–reperfusion injury^5^. Reactive oxygen species generated during this process might activate neutrophils and inflammatory mediators, leading to pulmonary capillary endothelial injury and the development of atelectasis and pulmonary oedema^6^. Elderly patients are particularly vulnerable to PPCs because of age-related structural and functional decline in the respiratory system, which increases susceptibility to lung injury compared with younger individuals^7^. These factors together may increase the risk of PPCs in elderly patients undergoing laparoscopic surgery in the Trendelenburg position.

Optimisation of intraoperative mechanical ventilation is an important part of perioperative lung protection and may influence the risk of PPCs^8^. In clinical practice, the two ventilation modes most commonly used are volume-controlled ventilation (VCV) and pressure-controlled ventilation (PCV). VCV delivers a preset tidal volume (VT) with a constant inspiratory flow. Under conditions of reduced pulmonary compliance or increased airway resistance, however, this flow pattern may be accompanied by higher peak inspiratory pressure (Ppeak), which could increase the risk of barotrauma and ventilator-induced lung injury (VILI). By comparison, PCV delivers ventilation at a set inspiratory pressure with a decelerating inspiratory flow pattern, although hypoventilation may occur when pulmonary compliance is markedly reduced^9,10^.

Previous investigations have yielded conflicting results. Some studies found that PCV reduced Ppeak and improved dynamic compliance (Cdyn) compared with VCV, but observed no significant differences in other respiratory parameters or clinical outcomes^11,12^. Others reported that PCV improved intraoperative oxygenation and reduced airway pressures^13^. A retrospective observational study indicated a higher incidence of PPCs in patients ventilated with PCV compared to VCV^14^. More recent research in patients undergoing thoracoscopic lung resection suggested that the choice of ventilation mode did not significantly influence the incidence of PPCs^15^. To date, there is a paucity of studies specifically investigating PPCs in the context of laparoscopic surgery performed in the Trendelenburg position. Consequently, no definitive conclusion exists regarding the optimal ventilation mode for this specific surgical and patient population.

Therefore, this randomized controlled trial aimed to evaluate the impact of PCV versus VCV on the incidence of PPCs in elderly patients undergoing laparoscopic surgery in the Trendelenburg position, with the goal of identifying an optimal ventilation strategy to improve postoperative outcomes.

## Materials and methods

### Study design and population

This clinical trial was designed as a prospective, randomized, controlled, and assessor-blinded study, carried out at the First Affiliated Hospital of Chongqing Medical University. Ethical approval for the study was granted by the Ethics Committee of the First Affiliated Hospital of Chongqing Medical University (CY2024-217-02), and the trial was registered at the Chinese Clinical Trial Registry (ChiCTR2400094228) before patient enrollment, with the registration date being December 19, 2024. This study adhered to the CONSORT statement, the Declaration of Helsinki, and relevant institutional and national requirements. Written informed consent was obtained from each participant, or from a legal guardian when applicable, before study enrolment.

Inclusion criteria were: (1) age 60–80 years; (2) American Society of Anesthesiologists (ASA) physical status II–III; (3) scheduled for elective laparoscopic surgery requiring the Trendelenburg position; (4) anticipated surgery duration > 2 h. Exclusion criteria were: (1) body mass index (BMI) > 28 kg/m²; (2) preoperative chest computed tomography (CT) indicating pulmonary infection, atelectasis, pleural effusion, or pneumothorax; (3) severe cardiac dysfunction (New York Heart Association class ≥ III); (4) severe hepatic impairment (Child-Pugh classification C); (5) chronic kidney disease (CKD) stage ≥G4; (6) contraindication to arterial catheterization.

### Randomization and Blinding

Patients were randomly assigned in a 1:1 ratio to the PCV or VCV group using a computer-generated allocation sequence. Group assignments were concealed in sequentially numbered, opaque, sealed envelopes, opened by a researcher not involved in the trial. The attending anesthesiologist, who was responsible for intraoperative management and ventilator setting, was aware of the group assignment. Outcome assessors evaluating PPCs during the postoperative ward stay were strictly blinded to the intraoperative ventilation mode. To preserve blinding throughout postoperative care, specific ventilator settings and group allocation details were systematically omitted from standard postoperative handover documents. Study data were collected on dedicated case report forms and entered separately into pre-/intraoperative and postoperative databases. The two datasets were combined only after completion of the study.

### Intervention and intraoperative management

Standard monitoring was applied upon arrival in the operating room. Before induction, an arterial catheter was placed under local anesthesia for continuous blood pressure monitoring. Patients received midazolam (0.05 mg/kg), propofol (2 mg/kg), sufentanil (0.5 µg/kg), and vecuronium (0.15 mg/kg) for induction of anesthesia. Anaesthesia was maintained with continuous propofol and remifentanil infusions combined with sevoflurane inhalation, with a target minimum alveolar concentration of 0.7–1.0 and a bispectral index of 40–50. Pneumoperitoneum pressure was maintained at 12 mmHg, and patients were positioned at 30° Trendelenburg. Temperature was maintained above 36 °C using warmed fluids and forced-air warming. After operation, patients were transferred to the post-anaesthesia care unit. Multimodal analgesia was provided to maintain a numerical rating scale score < 4.

After tracheal intubation, patients were ventilated in the assigned mode (PCV or VCV) with a Dräger Atlan A300 anesthesia machine. Ventilator settings followed a lung-protective strategy: tidal volume (VT) 6–8 mL/kg predicted body weight, positive end-expiratory pressure (PEEP) 5 cmH₂O, fraction of inspired oxygen (FiO₂) 0.5, inspiratory-to-expiratory ratio 1:2, and respiratory rate adjusted to maintain end-tidal carbon dioxide (ETCO₂) at 30–40 mmHg. In the PCV group, inspiratory pressure was adjusted according to the delivered VT. After pneumoperitoneum and Trendelenburg positioning, inspiratory pressure was increased promptly and then titrated throughout surgery as needed to maintain VT within 6–8 mL/kg and to avoid hypoventilation. If peak inspiratory pressure (Ppeak) reached 35 cmH₂O, VT was readjusted within the target range. At four predefined intraoperative time points (T1–T4), plateau pressure (Pplat) was measured with a 0.5 s inspiratory hold, and the previous ventilator settings were then restored. In the PCV group, inspiratory pressure was initially set at 12 cmH₂O and adjusted in 1–2 cmH₂O increments according to the delivered VT. Following pneumoperitoneum and Trendelenburg positioning, inspiratory pressure was further increased in 2 cmH₂O increments as required to maintain VT within the target range.

### Outcome measures

The primary endpoint was a composite measure of postoperative pulmonary complications arising within the first 3 postoperative days, classified according to the European Perioperative Clinical Outcome (EPCO) criteria^16^. The components of this endpoint were respiratory infection, respiratory failure, pleural effusion, atelectasis, pneumothorax, bronchospasm, and aspiration pneumonitis (Table 1). Patients were evaluated daily by two trained researchers during this period using clinical symptoms, physical examination findings, laboratory results, and available imaging. Postoperative lung ultrasound was performed routinely, and additional radiological examinations were obtained only when clinically indicated.

**Table 1:** Diagnostic criteria for postoperative pulmonary complications (based on EPCO definitions).

Postoperative pulmonary complications	Diagnostic criteria
Respiratory infection	Suspected respiratory system infection treated with antibiotics and meeting at least one of the following: new or changed cough/sputum; imaging showing pulmonary infection manifestations; fever; white blood cell count exceeding 12 × 10^9^/L.
Respiratory failure	PaO_2_ < 60 mmHg when breathing air, PaO_2_/FiO_2_ < 300 mmHg or SpO_2_ < 90%, and requiring oxygen therapy.
Pleural effusion	Diagnosis by imaging examination.
Atelectasis	Diagnosis by imaging examination.
Pneumothorax	Diagnosis by imaging examination.
Bronchospasm	Wheezing was auscultated during expiration, necessitating treatment with bronchodilators.
Aspiration pneumonitis	Acute lung injury after the inhalation of gastric contents.

Secondary outcomes comprised haemodynamic variables (heart rate and mean arterial pressure), respiratory mechanics (Ppeak, Pplat, driving pressure, Cdyn, dead-space fraction, RR, VT, and ETCO₂), and arterial blood gas measurements (pH, PaO₂, PaCO₂, lactate, oxygenation index, and alveolar–arterial oxygen gradient). All variables were recorded at the predefined time points (T0–T4) described above, with the following exceptions: respiratory mechanics were not measured at T0 because patients had not yet undergone mechanical ventilation, and arterial blood gas measurements were not performed at T1 because T1 was very close in time to T0 and baseline data had already been obtained at T0.

Ventilatory efficiency was evaluated using the dead-space fraction (VD/VT) and PaCO₂ under conditions of constant minute ventilation. VD/VT was derived according to the Enghoff equation: VD/VT = (PaCO₂ − ETCO₂)/PaCO₂. In addition, other calculation formulas include: driving pressure (ΔP) = Pplat – PEEP, Cdyn = VT / (Pplat – PEEP), oxygenation index (OI) = PaO₂/FiO₂, alveolar–arterial oxygen gradient [P(A-a)O₂] = (PB – PH₂O) × FiO₂ – PaCO₂ / R – PaO₂, In the above formula, PB (atmospheric pressure) = 760 mmHg, PH₂O (saturated water vapor pressure at room temperature) = 47 mmHg and R (respiratory quotient) = 0.8.

### Sample size and statistical analysis

The sample size was calculated using PASS (Power Analysis and Sample Size Software, Version 15.0; NCSS, Kaysville, UT, USA). The software is available at https://www.ncss.com/software/pass/. Previous literature indicated a PPC incidence of 13.0% for PCV group, compared to 39.1% for VCV group^17^. Based on these rates, and setting α = 0.05 with 80% power, we determined that 41 participants were needed in each group. Accounting for a 20% dropout rate, at least 52 patients per group (104 total) were targeted.

All statistical analyses were carried out with SPSS version 26.0 (IBM, Armonk, NY, USA). The primary endpoint was compared using the chi-square test or Fisher’s exact test where appropriate. Distribution of continuous variables was examined with the Shapiro–Wilk test. Normally distributed variables are reported as mean (standard deviation) and were analysed with repeated-measures analysis of variance or the independent-samples t-test. Bonferroni correction was used for multiple testing. Non-normally distributed variables are reported as median (interquartile range) and were analysed with generalized estimating equations or the Mann–Whitney U test, as appropriate. Categorical data were analysed with the chi-square test or Fisher’s exact test. Statistical significance was set at a two-sided P value of < 0.05.

## Results

### Study population

**Fig. 1:**
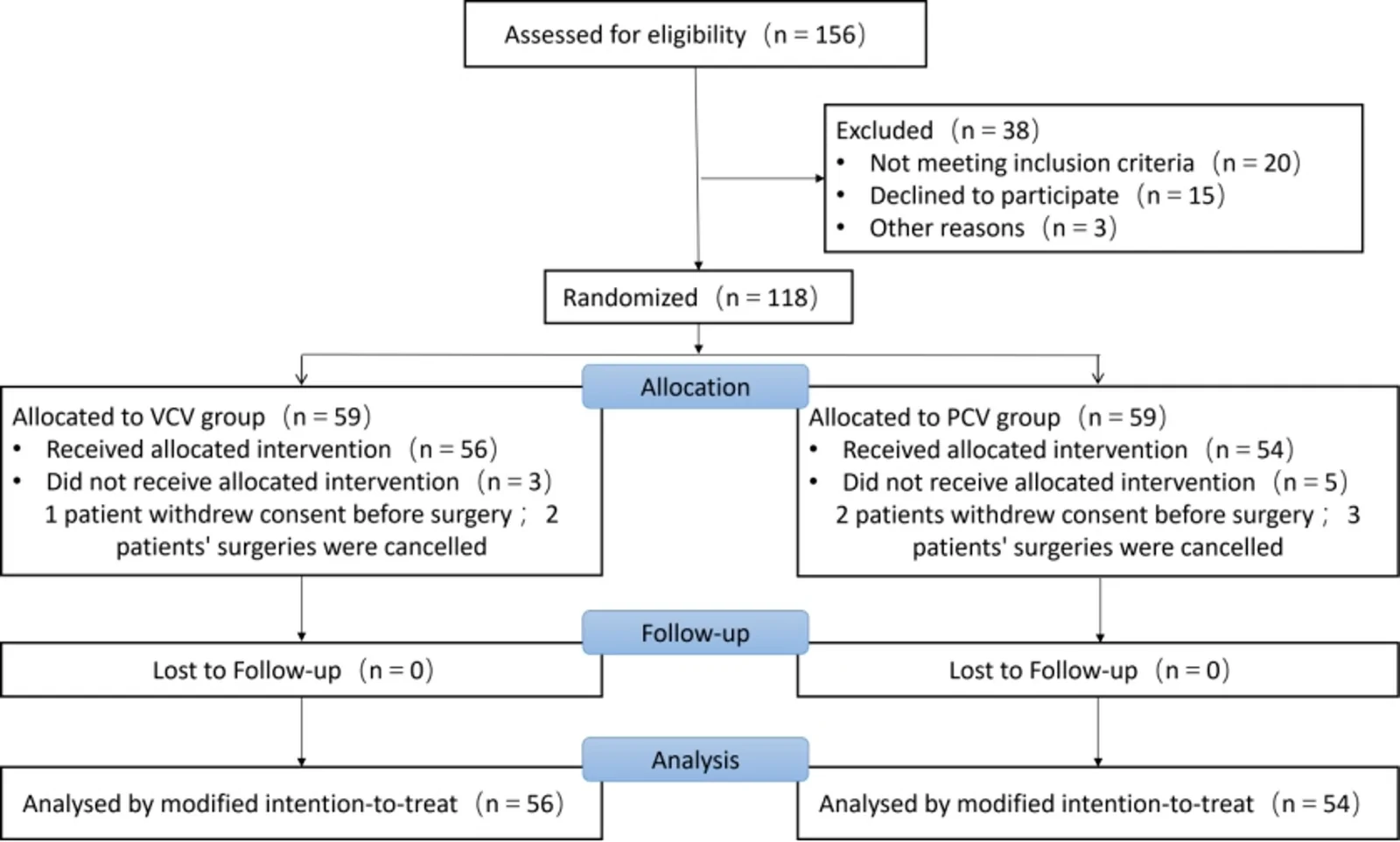
CONSORT flow diagram of patient enrollment, randomization, and follow-up.

Between December 2024 and October 2025, 156 patients were assessed for eligibility. This study used a modified intention-to-treat (mITT) analysis, 118 were randomized (59 per group). The intervention was not administered to five patients in the PCV group (two consent withdrawals, three surgery cancellations) and three in the VCV group (one consent withdrawal, two cancellations). Thus, 110 patients (54 PCV, 56 VCV) were included in the analysis (Fig. 1). No serious adverse events or unintended effects were observed in either group during the study period. The trial was terminated as planned after completion of recruitment and follow-up.

### Baseline characteristics and intraoperative data

**Fig. 2:**
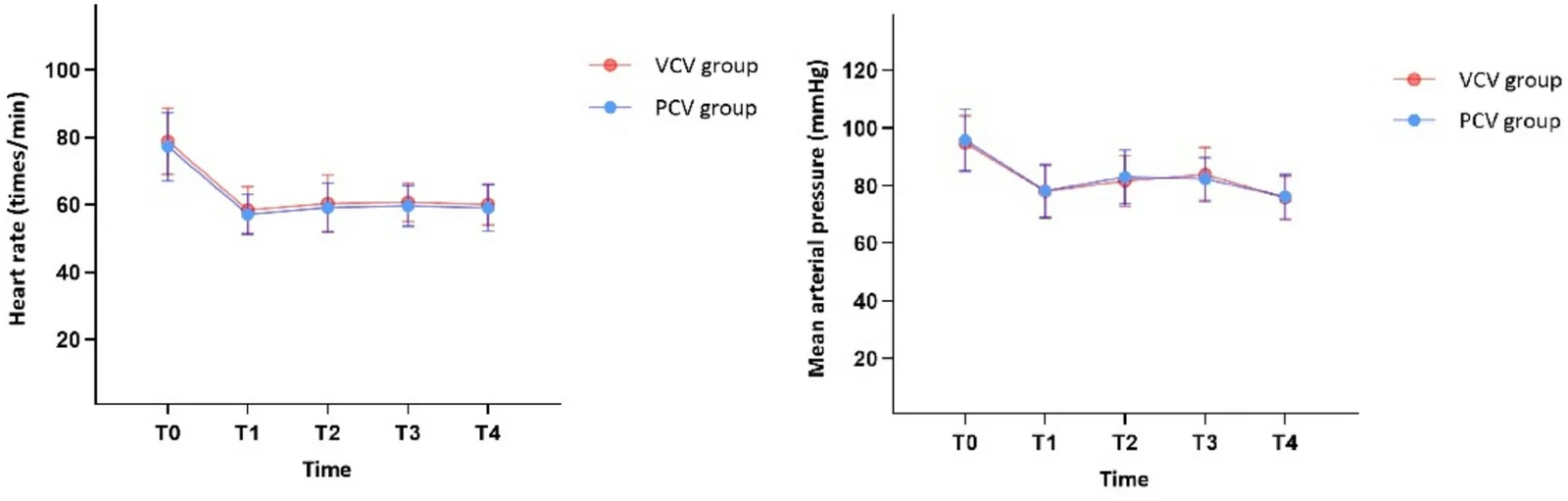
Intraoperative heart rate and mean arterial pressure at different time points. T0, before anesthesia induction; T1, 10 min after tracheal intubation in the supine position without pneumoperitoneum; T2 and T3, 30 and 60 min after establishing pneumoperitoneum and the Trendelenburg position; T4, at the end of surgery after returning to the supine position; PCV, pressure-controlled ventilation; VCV, volume-controlled ventilation.

Baseline demographics and clinical characteristics were well-balanced across the two groups (Table 2). Intraoperative fluid administration, blood loss, urine output, heart rate, and mean arterial pressure did not differ significantly between groups (Table 3; Fig. 2).

**Table 2:** Baseline characteristics of the study population.

	PCV group (*n* = 54)	VCV group (*n* = 56)	*P*-value
Age (years)	68.3 ± 5.1	68.5 ± 5.3	0.780
BMI (kg/m^2^)	23.4 ± 2.0	23.0 ± 2.5	0.352
Sex			
Male	8 (14.8)	6 (10.7)	0.519
Female	46 (85.2)	50 (89.3)	
ASA physical status			
II	38 (70.4)	41 (73.2)	0.741
III	16 (29.6)	15 (26.8)	0.460
Comorbidity			
Hypertension	22 (40.7)	19 (33.9)	0.724
Diabetes	8 (14.8)	7 (12.5)	0.733
Coronary artery disease	3 (5.6)	4 (7.1)	0.204
Type of surgery			
Gynecological	40 (74.1)	47 (83.9)	
Colorectal	14 (25.9)	9 (16.1)	
Duration of surgery (min)	155.0 [130.0-192.5]	160.0 [135.0-196.3]	0.303
Duration of laparoscopy (min)	130.0 [105.0-165.0]	135.5 [111.3-177.8]	0.412
Duration of anesthesia (min)	195.5 [174.8-242.5]	200.0 [181.3-248.8]	0.478

Data are presented as mean ± standard deviation, number (percentage), or median [interquartile range].

*PCV* pressure-controlled ventilation; *VCV* volume-controlled ventilation; *BMI* body mass index; *ASA* American Society of Anesthesiologists.

**Table 3:** Intraoperative fluid management and blood loss.

	PCV group (*n* = 54)	VCV group (*n* = 56)	P-value
Crystalloid infusion volume (ml)	1100 [1087.5–1100]	1100 [1100-1137.5]	0.588
Colloid infusion volume (ml)	500 [0-500]	500 [0-500]	0.940
Total infusion volume (ml)	1600 [1100–1600]	1600 [1100–1600]	0.788
Blood loss (ml)	50 [20–100]	80 [50–100]	0.357
Urine volume (ml)	350 [200–600]	475 [300–700]	0.258

Data are presented as median [interquartile range].

*PCV* pressure-controlled ventilation; *VCV* volume-controlled ventilation.

### Primary and secondary outcomes

#### Primary outcome

Within 3 postoperative days, PPCs developed in 7 of 54 patients (13.0%) in the PCV group and 18 of 56 patients (32.1%) in the VCV group (χ²=5.758, *P* = 0.016), yielding a relative risk of 0.403 (95% CI, 0.183–0.888). The absolute risk reduction was 19.1% (95% CI, 5.5–32.7%), corresponding to a number needed to treat of 6 (95% CI, 3–19). Across the whole cohort, atelectasis was the most frequent PPC (16/110, 14.5%), followed by respiratory infection (11/110, 10.0%) and respiratory failure (3/110, 2.7%). No significant differences were found between groups for any individual PPC subtype (Table 4).

**Table 4:** Incidence of postoperative pulmonary complications within 3 days.

	PCV group (*n* = 54)	VCV group (*n* = 56)	*P*-value
PPCs within 3 days	7 (13.0)	18 (32.1)	0.016
Respiratory infection	3 (5.6)	8 (14.3)	0.127
Atelectasis	5 (9.3)	11 (19.6)	0.123
Respiratory failure	1 (1.9)	2 (3.6)	1.000
Pleural effusion	0 (0.0)	1 (1.8)	1.000
Pneumothorax	0	0	
Bronchospasm	0	0	
Aspiration pneumonia	0	0	

Data are presented as number (percentage).

*PPCs* postoperative pulmonary complications; *PCV* pressure-controlled ventilation; *VCV* volume-controlled ventilation.

#### Secondary outcomes

**Fig. 3:**
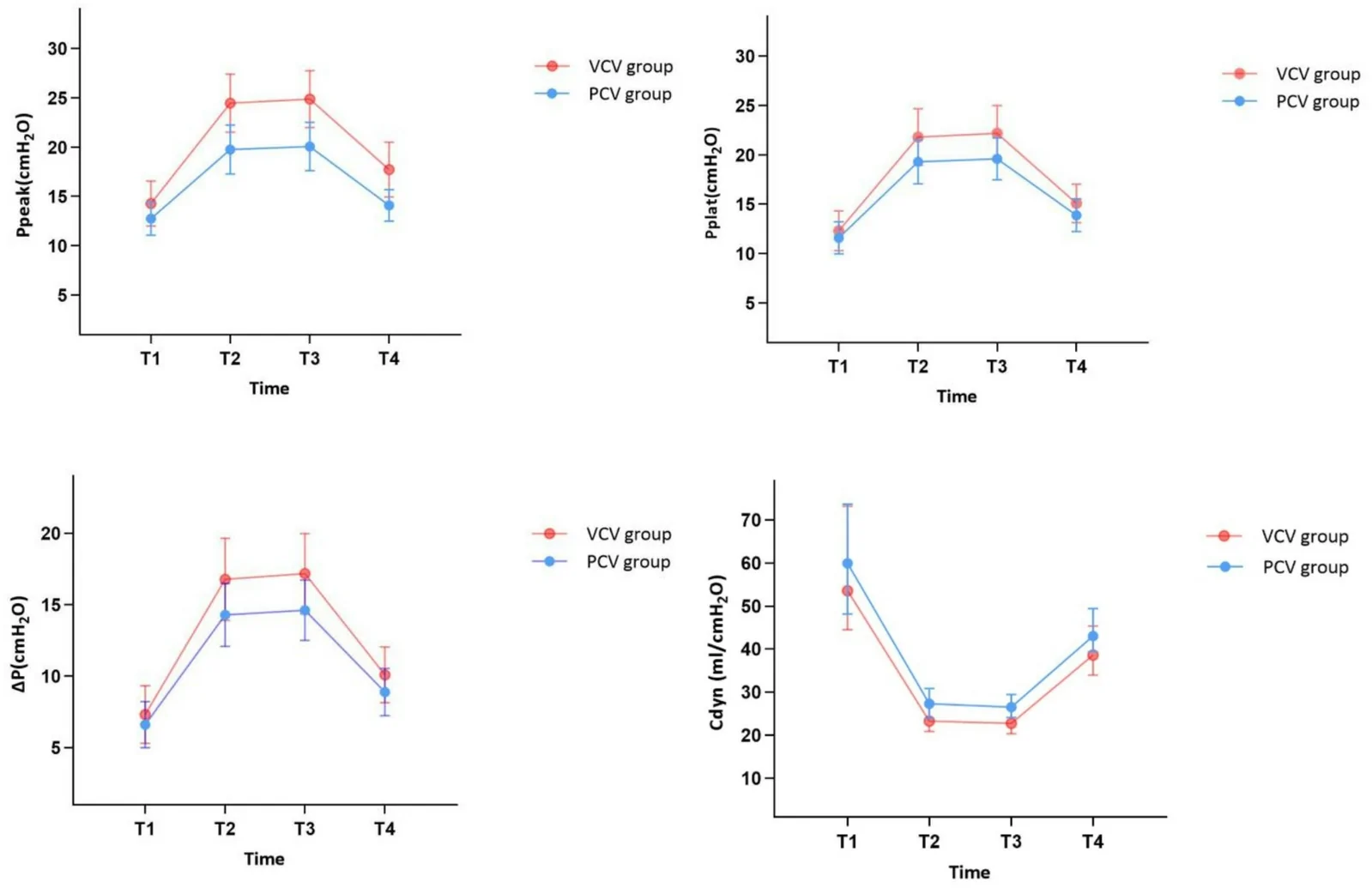
Comparison of intraoperative respiratory mechanics at different time points. T1, 10 min after tracheal intubation in the supine position without pneumoperitoneum; T2 and T3, 30 and 60 min after establishing pneumoperitoneum and the Trendelenburg position; T4, at the end of surgery after returning to the supine position; PCV, pressure-controlled ventilation; VCV, volume-controlled ventilation.

Compared with the VCV group, the PCV group exhibited significantly lower Ppeak, Pplat, and ΔP at all intraoperative time points (T1–T4), along with higher Cdyn and lower VD/VT at T2–T4 (*P* < 0.05 for all). No significant between-group differences were observed in RR or VT. ETCO₂ was significantly lower in the PCV group only at T4(Fig. 3) (Table 5).

**Table 5:** Intraoperative respiratory parameters.

	Group	T1	T2	T3	T4	*P*-value
Ppeak(cmH_2_O)	PCV	12.8 ± 1.7^a^	19.8 ± 2.5^a^	20.1 ± 2.5^a^	14.1 ± 1.6^a^	< 0.001
	VCV	14.3 ± 2.3	24.5 ± 2.9	24.9 ± 2.9	17.1 ± 2.8	
Pplat (cmH_2_O)	PCV	11.6 ± 1.6^a^	19.3 ± 2.2^a^	19.6 ± 2.1^a^	13.9 ± 1.7^a^	< 0.001
	VCV	12.3 ± 2.0	21.8 ± 2.9	22.2 ± 2.8	15.1 ± 2.0	
ΔP (cmH_2_O)	PCV	6.6 ± 1.6^a^	14.3 ± 2.2^a^	14.6 ± 2.1^a^	8.9 ± 1.7^a^	< 0.001
	VCV	7.3 ± 2.0	16.8 ± 2.9	17.2 ± 2.8	10.1 ± 2.0	
Cdyn (ml/cmH_2_O)	PCV	60 [48–74]	27 [24–31]^a^	27 [24–29]^a^	43 [39–49]^a^	0.024
	VCV	54 [45–73]	23 [21–27]	23 [20–27]	39 [34–45]	
VD/VT (%)	PCV	/	11 ± 5^a^	12 ± 6^a^	11 ± 6^a^	0.007
	VCV	/	13 ± 6	15 ± 6	14 ± 7	
ETCO_2_ (mmHg)	PCV	32 [30–34]	35 [33–37]	35 [34–37]	34 [32–35]^a^	0.305
	VCV	32 [31–34]	35 [34–37]	35 [33.25-37]	35 [33–37]	
RR(breaths/min)	PCV	12 [12–12]	15 [14–15]	15 [14–15]	13 [12–13]	0.278
	VCV	12 [12–13]	15 [14–15]	15 [14–15]	13 [12–13]	
VT(ml)	PCV	388 ± 36	393 ± 40	390 ± 42	391 ± 41	0.169
	VCV	397 ± 30	401 ± 31	402 ± 31	397 ± 29	

Data are shown as mean ± standard deviation or median [interquartile range].

*Ppeak* peak inspiratory pressure; *Pplat* plateau pressure; *ΔP* driving pressure; *Cdyn* dynamic lung compliance; *VD/VT* physiological dead space to tidal volume ratio; *ETCO*_2_, end tidal CO_2_; *RR* respiratory rate; *VT* tidal volume; *PCV* pressure-controlled ventilation; *VCV* volume-controlled ventilation.

^a^*p* < 0.05 compared with VCV.

Regarding arterial blood gases, PaCO₂ was significantly lower and pH significantly higher in the PCV group at T2, T3, and T4 (^a^*P*< 0.05). No significant differences were found in PaO₂, oxygenation index, alveolar-arterial oxygen gradient, or lactate levels (Table 6).

**Table 6:** Intraoperative arterial blood gas measurements.

	Group	T0	T2	T3	T4	*P*-value
PaO_2_(mmHg)	PCV	86 ± 8	225 ± 34	223 ± 32	226 ± 35	0.304
	VCV	86 ± 8	219 ± 42	214 ± 36	220 ± 42	
OI(mmHg)	PCV	411 ± 36	450 ± 69	446 ± 63	454 ± 73	0.296
	VCV	409 ± 39	438 ± 84	428 ± 73	441 ± 85	
P(A-a)O₂(mmHg)	PCV	17 ± 7	82 ± 34	83 ± 31	83 ± 35	0.458
	VCV	17 ± 8	87 ± 41	91 ± 36	85 ± 42	
PaCO_2_(mmHg)	PCV	37.17 ± 3.21	39.50 ± 2.97^a^	40.22 ± 2.72^a^	38.15 ± 3.74^a^	0.002
	VCV	37.38 ± 3.66	40.63 ± 2.96	41.36 ± 2.86	40.75 ± 3.19	
pH	PCV	7.42 ± 0.03	7.37 ± 0.03^a^	7.36 ± 0.04^a^	7.37 ± 0.05^a^	0.003
	VCV	7.41 ± 0.04	7.36 ± 0.05	7.34 ± 0.04	7.34 ± 0.05	
Lac	PCV	1.0[0.8–1.3]	0.7[0.6–0.8]	0.7[0.6–0.8]	0.7[0.6–0.8]	0.617
	VCV	0.9[0.7–1.1]	0.7[0.6–0.9]	0.7[0.5–0.8]	0.7[0.5–0.9]	

Data are shown as mean ± standard deviation or median [interquartile range].

PaO_2_, arterial partial pressure of oxygen; OI, oxygenation index; P(A-a)O₂, alveolar–arterial oxygen gradient; PaCO_2_, arterial partial pressure of carbon dioxide; pH, potential of hydrogen; Lac, lactate; PCV, pressure-controlled ventilation; VCV, volume-controlled ventilation.

^a^*p* < 0.05 compared with VCV.

## Discussion

This prospective randomized controlled trial showed that pressure-controlled ventilation (PCV) resulted in fewer postoperative pulmonary complications (PPCs) than volume-controlled ventilation (VCV) among elderly patients undergoing laparoscopic surgery in the Trendelenburg position. Furthermore, PCV was associated with improved respiratory mechanics and better ventilatory efficiency, without compromising oxygenation.

Mechanical ventilation can induce ventilator-induced lung injury (VILI)^18^. During laparoscopic surgery in the Trendelenburg position, pneumoperitoneum restricts diaphragmatic excursion, reduces pulmonary compliance, and increases airway pressures—effects that are exacerbated by the head-down position^3,4,9^. The constant inspiratory flow of VCV in this setting may generate elevated airway pressures, potentially aggravating VILI and increasing PPC risk. Given that elderly patients represent a high-risk population for PPCs^7,19^, this study sought to determine whether PCV could improve clinical outcomes in this vulnerable group.

Consistent with previous reports^17,20^, the PCV group had lower peak and plateau airway pressures and higher dynamic compliance than the VCV group throughout surgery, including after release of pneumoperitoneum. Driving pressure (ΔP) has been closely associated with postoperative survival and the development of postoperative pulmonary complications (PPCs)^21,22^. Douville and colleagues reported that prolonged exposure to a ΔP > 16 cm H₂O was associated with an increased risk of PPCs^23^. In the present study, ΔP was also significantly lower in the PCV group. Notably, mean ΔP remained below 16 cm H₂O in the PCV group, whereas this threshold was exceeded in the VCV group during pneumoperitoneum. This difference may therefore be clinically relevant, rather than merely statistically significant. Taken together, these findings suggest that PCV provides more favourable respiratory mechanics in this setting. A possible explanation is that PCV limits inspiratory airway pressure and thereby attenuates the rise in airway pressures associated with pneumoperitoneum and Trendelenburg positioning. In addition, the decelerating inspiratory flow pattern of PCV may promote more even distribution of ventilation and improve gas delivery to dependent lung regions that are particularly susceptible to collapse under these conditions.

In the present study, VD/VT was also lower in the PCV group, in keeping with previous findings^17^. With minute ventilation maintained at a similar level between groups, PaCO₂ was lower in the PCV group, which may indicate more efficient ventilation. One possible explanation is improved recruitment of previously collapsed or poorly ventilated lung units with the decelerating flow pattern of PCV, resulting in more effective gas exchange. This is also consistent with the relatively higher pH in the PCV group. Because lactate concentration and fluid balance did not differ between groups, impaired tissue perfusion or differences in intravascular volume status are unlikely to account for these acid–base findings. The clinical importance of these physiological differences, however, remains unclear. Although PCV was associated with improved respiratory mechanics and ventilatory efficiency, whether this translates into better patient-centred outcomes, including shorter hospital stay or lower mortality, remains uncertain. Further studies are required to clarify the clinical relevance of these findings.

Unlike some reports in obese patients^24^, we did not observe significant differences in oxygenation, including PaO₂, oxygenation index, or alveolar–arterial oxygen gradient, between the two ventilation modes. This may be explained by the use of identical FiO₂ and PEEP in both groups and by the relatively preserved baseline pulmonary function of the study population. In this setting, the physiological effects of PCV therefore appeared to be greater on respiratory mechanics and CO₂ clearance than on oxygenation. Both groups received the same lung-protective ventilation strategy (VT 6–8 mL/kg, PEEP 5 cmH₂O, FiO₂ 0.5). Although the PCV group had lower driving pressure and PaCO₂, the absolute differences were modest, and their clinical significance remains unclear. In older patients, age-related chest wall stiffening and reduced lung compliance may increase vulnerability to mechanical stress, so that even small reductions in driving pressure could theoretically reduce dynamic lung stretch and limit ventilator-induced lung injury. Even so, the present study does not allow firm conclusions as to whether these physiological differences accounted for the observed clinical results, and their patient-centred significance requires further study.

PPCs were diagnosed using a graded protocol. All patients underwent routine postoperative lung ultrasonography, and any abnormal ultrasonographic findings or clinical symptoms prompted further imaging with chest radiography or computed tomography. Ultrasound examinations were performed by two trained investigators who were blinded to group allocation and followed a standardized protocol described previously. Although this approach reduced the risk of differential ascertainment between groups, routine ultrasonography may have increased detection of subclinical events, particularly mild atelectasis, thereby giving greater weight to less severe components of the composite endpoint.

Accordingly, although the overall incidence of composite PPCs was significantly lower in the PCV group than in the VCV group, no individual PPC component differed significantly between groups. This finding should be interpreted with caution. First, the study was powered for the composite endpoint rather than for individual complications and was therefore likely underpowered to detect differences in relatively infrequent events. Our sample size calculation was based on the study by Choi et al.^17^, in which the reported PPC rates were 13.0% in the PCV group and 39.1% in the VCV group. In the present study, however, the observed PPC rate in the VCV group was 32.1%, resulting in a smaller between-group difference than anticipated and, consequently, lower statistical power. This discrepancy may be related to differences in patient population and perioperative ventilation practice, including the routine use of 5 cmH₂O PEEP in our cohort. Second, as with any composite endpoint, a significant overall effect does not necessarily indicate benefit for each individual component, but may instead reflect the cumulative effect of several small, directionally consistent, yet individually non-significant differences. In our data, no clear trend favouring PCV was observed for major respiratory events such as respiratory failure, pleural effusion, pneumothorax, or aspiration pneumonia. Rather, the reduction in the composite endpoint may have been driven largely by fewer cases of atelectasis and pneumonia, including mild events detected by routine ultrasonography. Therefore, although PCV was associated with a lower composite PPC rate, these findings do not establish that PCV prevents any specific pulmonary complication, and whether the physiological advantages of PCV translate into meaningful patient-centred outcomes remains uncertain. Larger studies powered for individual pulmonary endpoints are needed to clarify the effects of PCV on specific postoperative respiratory complications.

### Limitations

Several limitations merit consideration. This single-centre trial included only older patients undergoing laparoscopic surgery in the Trendelenburg position with a fixed pneumoperitoneum pressure of 12 mmHg, which may limit generalisability. The study may also have been underpowered for individual PPC components and secondary outcomes, as the observed event rate in the VCV group was lower than anticipated from the data used for sample size estimation, possibly reflecting the consistent use of lung-protective ventilation in both groups. The modest sample size and 3-day follow-up further limited the assessment of infrequent events and longer-term outcomes. In addition, interpretation of the primary endpoint is constrained by the use of a composite PPC outcome, as no individual component differed significantly between groups. Although outcome assessors were blinded and applied standardised EPCO criteria, the attending anaesthetist could not be blinded to group allocation. Airway pressure was measured at the ventilator side following standard clinical practice; however, due to the high resistance of the endotracheal tube and connectors^25,26^, the actual differences between the two ventilation modes at the alveolar level may be masked, and our proximal measurements may not directly reflect alveolar changes. Finally, radiological confirmation was obtained only when clinically indicated; however, all patients underwent routine postoperative lung ultrasonography, which should have reduced differential ascertainment between groups, albeit with greater detection of subclinical abnormalities.

## Conclusions

In this exploratory study of older patients undergoing laparoscopic surgery in the Trendelenburg position, PCV was associated with better respiratory mechanics, more efficient ventilation, and a lower composite rate of PPCs within the first 3 postoperative days than VCV. However, PCV did not improve oxygenation or reduce any individual pulmonary complication. Given the modest sample size, short follow-up, and reliance on a composite endpoint, these findings should be interpreted cautiously and do not support a definitive clinical recommendation. Further multicentre studies with longer follow-up and adequate power for individual complications are required to clarify whether these physiological effects are associated with clinical benefit.

## Data Availability

The datasets used and/or analyzed during the current study are available from the corresponding author upon reasonable request.
